# 电视硬质气管镜在大气道狭窄治疗中的应用

**DOI:** 10.3779/j.issn.1009-3419.2011.04.12

**Published:** 2011-04-20

**Authors:** 洪武 王, 云芝 周, 冬妹 李, 楠 张, 珩 邹, 晶 李, 素娟 梁

**Affiliations:** 100028 北京，煤炭总医院肿瘤微创治疗中心 Minimal Invasive Tumor Terapy Center, Meitan General Hospital, Beijing 100028, China

**Keywords:** 气道狭窄, 恶性肿瘤, 支气管镜, Airway obstruction, Malignant tumor, Bronchoscopy

## Abstract

**背景与目的:**

大气道狭窄是内、外科和麻醉科都较难处理的重大疾病, 如不及时处理易引起窒息。本文旨在探讨应用电视硬质气管镜消除气道狭窄的可行性、有效性和安全性。

**方法:**

回顾分析2007年8月27日-2010年9月30日收治的194例大气道狭窄病例（平均年龄（57.5±1.3）岁，其中男性140例，女性54例；恶性气道狭窄145例，良性气道狭窄49例）在全身麻醉支持下经口插入硬质镜，连接高频通气，结合电子支气管镜对声门部、气管内及支气管内狭窄采用电圈套器、冷冻、氩等离子体凝固（argon plasma coagulation, APC）等综合治疗措施进行治疗的情况。

**结果:**

194例患者共接受了325次硬质镜检查，平均每例患者接受1.6次操作，硬质镜检查占所有气管镜检查的21.3%（325/1, 525）。气道内肿瘤包括原发肿瘤76例，转移性肿瘤69例。良性狭窄最常见病因为瘢痕狭窄，其次为良性肿瘤、原发性肉芽组织增生、异物、气管软化和复发性多发性软骨炎。硬质镜首次治疗后气道狭窄程度均明显下降，其中支气管的下降程度要大于主气管。首次治疗后KPS明显升高，气促指数明显下降。硬质镜下取出气管支架26个，放置气管支架13个。硬质镜治疗比较安全，术中死亡1例。

**结论:**

硬质镜治疗大气道狭窄较快速、有效、安全，能提高患者生存质量。

## Abstract (en)

**Background and objective:**

Trentment of critical airway stenoses remains a formidable challenge to surgeons and anesthiologists. Suffocate is a leading cause of death if the patients with airway diseases were not treated properly. The perpose of this study is to explore the feasibility, safety and efciency using video-assisted rigid bronchoscopes (VARB) in the treatment of central airway stenoses.

**Methods:**

From August 27, 2007 to September 30, 2010, 194 patients (140 men and 54 women, mean age 57.5±1.3 years) with airway stenoses (145 with malignant and 49 with benign stenoses) were retrospectively reviewed underwent VARB treatment in our hospital. All the procedures were carried out under general anesthesia with high frequency jet ventilation. Afer the VARB was placed in the main trachea through the mouth, electric bronchoscope was combined to examine the lesions. Various methods for controlling the airway have been established to solve this problem, such as the electric loop, cryoprobe or argon plasma coagulation (APC) and stents.

**Results:**

T325 procedures were accomplished in all the 194 cases. Every patients received 1.6 procedures of VARB. VARB occupied 21.3% (325/1, 525) in all bronchoscopic procedures. Among the patients, there were 76 cases with primary airway tumor and 69 with secondary malignant tumor, 49 with benign airway stenoses. Karnofsky performance status (KPS) and short breathless index were obviously improved afer the frst treatment of VARB. Improvement of bronchial stenoses was superior to that of tracheal stenoses. The effect of electasis treatment is beter in obstruction of main bronchus than that of segment bronchus. 26 airway stents were removed and 13 stents were successfully placed under VARB.

**Conclusion:**

VARB can be applied safely and effectively for the management of tracheobronchial stenoses. Quality of life was improved afer the treatment of VARB.

大气道狭窄是指发生于声门、主气管及主支气管的狭窄，往往引起患者明显的呼吸困难，随时有窒息的可能，特别是气道严重狭窄的病例，一般难以耐受局麻下支气管镜检查，但又需做紧急处理。近年来作者采取全麻下电视硬质气管镜（简称硬质镜）结合电子支气管镜，应用电圈套器、二氧化碳（CO_2_）冷冻、氩等离子体凝固（argon plasma coagulation, APC）等综合治疗措施治疗大气道狭窄，取得非常好的疗效，值得临床借鉴。

## 1 资料与方法

### 1.1 临床资料

2007年8月27日-2010年9月30日我院收治的194例大气道狭窄病例，年龄5岁-90岁，平均年龄（57.5 ±1.3）岁。恶性肿瘤145例，包括男性118例（平均年龄（61.0±1.5）岁），女性27例（平均年龄（62.4±3.1）岁）；原发肿瘤76例（肺癌72例，喉癌3例，下咽癌1例），转移性肿瘤69例（其中肺癌40例，食管癌15例，甲状腺癌5例，结肠癌3例，肾癌3例，肝癌、乳腺癌、胆管癌各1例）；有TNM分期的肿瘤病例Ib期3例，Ⅲa期10例，Ⅲb期42例，Ⅳ期80例。49例良性气道狭窄的病例，其中男性31例，女性18例，年龄6岁-78岁，平均（43.4± 3.0）岁，包括瘢痕狭窄21例，原发性肉芽肿增生9例，良性肿瘤10例，异物5例，气管软化3例，复发性多发性软骨炎1例。

### 1.2 治疗方法

#### 1.2.1 术前准备及麻醉

硬质镜操作均行全身麻醉，术前完善各项检查，如血常规、心电图、血氧饱和度、血气分析、肺功能、胸部X线和CT等，由麻醉师和临床医师进行术前评估和麻醉评估。麻醉前面罩吸氧，预氧合5 min-10 min。术前10 min静脉滴注阿托品0.5 mg或东莨菪碱0.3 mg，以抑制气道内过多的分泌物。术中需监测血氧饱和度、心电图、血压及呼吸运动等。麻醉诱导前5 min应用咪哒唑仑2 mg静注，随后静注芬太尼1 μg/kg-2 μg/kg，1%异丙酚1 mg/kg-2 mg/kg，然后给予肌松剂阿曲库铵0.5 mg/kg，待肌颤消失、下颌肌肉松弛后即可插入硬质镜。维持药物浓度为1%异丙酚1 mg/(kg·h)-2 mg/(kg· h)和瑞芬太尼0.1 μg/(kg·min)-0.2 μg/(kg·min)。

#### 1.2.2 硬质镜置入方法和机械通气

所用硬质镜为德国Karl Storz（Tutlingen）。患者平卧手术床上，在间接喉镜引导下或直视下插入硬质镜，接麻醉呼吸机，维持患者血氧饱和度在100%。声门部或声门下肿瘤，硬质镜前端斜面跨过声门即可，由助手固定硬质镜进行操作。

介入操作前换用高频喷射通气（频率40次/min-80次/min），连接三通管，在不停止呼吸机的情况下进行各种检查和治疗。若操作一段时间后，高频喷射通气不能维持足够的氧饱和度，可改用麻醉机，必要时用手动式球囊按压，使血氧饱和度维持在100%以上时，再继续进行操作。

#### 1.2.3 介入操作方法

通过硬质镜后端的操作孔进行各种操作，通常是结合电子支气管镜（所用设备为日本PENTAX-EPM 3500）进行APC、CO_2_冷冻等操作。

##### 1.2.3.1 APC

所用设备为德国产CESEL 3000型。将APC探针通过电子支气管镜活检孔伸出气管镜插入端（能见到探针标志为准），在距病灶0.5 cm以内时开始烧灼。APC输出功率为30 W-50 W，氩气流量为0.8 L/min-1.6 L/min。烧灼过程中不需停止吸氧，但以间断烧灼为宜（每次5s-10s左右），时间不能太长，并不断用活检钳取出碳化凝固的组织（碳化的组织易燃）。

##### 1.2.3.2 CO_2_冷冻

冷冻机采用北京库兰医疗设备有限公司生产的冷冻治疗仪K300型。软式可弯曲冷冻探头直径1.9 mm-2.3 mm，探针末端长度5 mm。冷源为液态CO_2_。将冰冻探头的金属头部放在肿瘤表面或推进到肿瘤内，冷冻5s-10s，使其周围产生最大体积的冰球，在冷冻状态下将探头及其粘附的肿瘤组织取出，必要时再插入探头，直至将腔内的肿瘤全部取出。冻取后如有出血，则结合APC止血。若将冰冻探头的金属头部放在病灶表面持续冷冻1 min-3 min，称为冻融。

##### 1.2.3.3 硬质镜下肿瘤铲除

对堵塞管腔50%以上的主气管内肿瘤，如果基底较宽，可在硬质镜下将肿瘤铲除，然后用光学活检钳或冷冻将铲下的肿瘤取出。如有出血，则用APC止血。

##### 1.2.3.4 电圈套器

电圈套器型号为南京微创公司生产。将电圈套器连接在高频电刀上。通过电子支气管镜的活检通道将电圈套器套扎在肿瘤上，然后启动高频电凝，将肿瘤切下，再使用光学活检钳或冷冻将切下的肿瘤取出。

### 1.3 统计分析方法

采用SPSS 11.0统计软件包，数据采用*t*检验分析，*P* < 0.05为有统计学差异。

## 2 结果

### 2.1 恶性肿瘤的治疗情况

#### 2.1.1 治疗效果

145例患者共进行硬质镜操作249例次，均一次性插入成功（4例次在间接喉镜引导下插入，245例次直接插入），每例平均进行（1.6±0.1）次。145例患者共接受了856次气管镜检查，每例平均检查5.9次，硬质镜的操作次数占29.1%。

多数病例气道病变以复合病变（两个部位以上）为主，占47.6%（69/145），其中以主气管内病变最多。硬镜治疗前和首次治疗后气道阻塞程度及气促指数的变化见表 1。硬镜治疗前以左下支气管阻塞程度最重，但两侧主支气管阻塞程度无明显差异。首次治疗后气道阻塞程度均明显下降（图 1），其中支气管的下降程度要优于主气管。首次治疗后KPS明显升高，气促指数明显下降。

**1:** 硬镜首次治疗前后恶性气道狭窄患者的病情变化 Changes of airway obstruction and clinical manifestations before and after the first rigid bronchoscopy in patients with malignant airway strictures

Observed index	*n*	Before	After	Changes
Trachea obstruction^☆^ (%)	164	59.2±1.4	15.4±0.1^*^	43.8±0.8
Right main bronchus obstruction (%)	53	72.1±3.6	14.4±1.8^*^	57.7±2.0
Intermedius bronchus obstruction (%)	27	78.8±5.1	11.9±4.3^*^	66.9±3.3
Left main bronchus obstruction (%)	79	69.3±3.3	12.0±1.4^*^	57.3±1.8
Left lower bronchus obstruction (%)	10	95.5±3.4	19.5±7.2^*^	76.0±4.0
Karnofsky physical score (KPS)	249	56.5±1.2	72.3±1.3^*^	15.8±1.1
Shortbreath score	249	3.2±0.1	1.6±0.1^*^	1.6±0.1
^☆^Obstruction (%) = (Normal airway size-the narrowest airway size)/Normal airway size×100%; ^*^P < 0.01 (comparison between the two groups of before and after therapy).				

**1:**
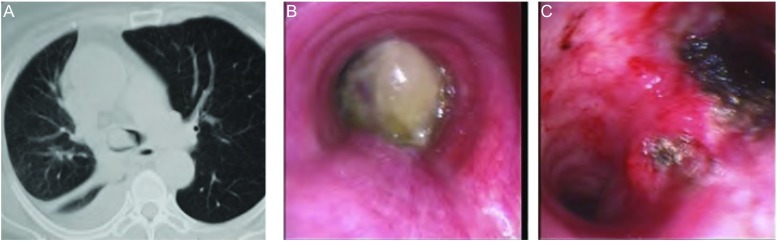
右肾癌切除术后右下肺及气管转移（女，64岁）。A：气管下段可见新生物，管腔堵塞2/3，伴右下肺不张、右胸腔积液；B：气管镜所见气管下端息肉样肿物堵塞管腔，隆突及双侧支气管开口未见；C：经硬质镜治疗后，腔内肿瘤大部分清除，管腔扩大，双侧支气管开口通畅。 Metastasis of right lung and trachea from kidney carcinoma after nephrotomy. A: 2/3 of lumen was obstructed by neoplasma in lower trachea accompanied with right lower lung etelectasis and pleural effusion; B: Lower trachea was incompletely obstructed by neoplasma in bronchoscopy. Carina and bilateral orificia of bronchus were not discovered; C: Most of tumor was removed, and endolumin expanded, and bilateral orificia of bronchus reopened after rigid brochoscopy.

肺不张的患者经硬质镜治疗后的效果见表 2。20例全肺不张的患者，经硬质镜治疗后全肺复张11例（55%），部分复张7例（35%），只有2例未能复张（肿瘤均位于4级以下支气管内）。7例右中下肺不张（肿瘤位于右中间段）经治疗后肺不张均消失。31例肺段不张的患者，经硬镜治疗后12例（38.7%）全部复张，7例（22.6%）部分复张，12例（38.7%）因肿瘤源于肺内，未能复张。全肺不张的治疗效果明显优于肺段不张。

**2:** 肺不张患者经硬质镜治疗后肺不张的变化 Changes of etelectasis in patients with pulmonary etelectasis after rigid bronchoscopy

Location of etelectasis	*n*	Complete remission	Partial remission	No remission
Right lung				
Whole	8	6	2	0
Superior lobe	9	4	2	3
Intermedius bronshus	7	7	0	0
Middle lobe	5	1	1	3
Inferior lobe	2	0	1	1
Left lung				
Whole	12	5	5	2
Superior lobe	5	3	0	2
Lingular lobe	3	0	1	2
Inferior lobe	8	4	2	2

在硬质镜下除清除肿瘤外，还取出气管支架5例，放置L型支架6例、植入放/化疗粒子6例、光动力治疗（PDT）3例。

#### 2.1.2 并发症及处理

包括门齿脱落2例（原有松动），术中大出血24例，其中23例经APC、喷洒止血药等血止，1例经抢救无效死亡。

249例次治疗过程中出现179例次（占71.9%）术中血氧饱和度低于90%，采取措施为暂停治疗、改接麻醉机快速直接充氧、手法控制呼吸直到血氧饱和度恢复至100%后继续治疗；治疗中有53例重症患者需要持续应用麻醉机快速直接充氧手法控制呼吸，直至肿瘤堵塞腔内的肿瘤清除后方改为高频喷射通气。

术中气管内着火5例。1例为肾癌气管转移，APC烧灼过程中出血较多，血氧饱和度在75%以下，通过硬质气管镜侧管置入细管给氧，并持续烧灼时点燃给氧管，将电子镜烧毁。另4例均在取气管内裸支架时，支架变形、扭曲，将气管堵塞，血氧饱和度持续下降，随即用APC连续烧灼，将支架烧断，因此引起气道内着火并烧毁软镜。着火后，应迅速撤出支气管镜和易燃物，然后对气道内进行冲洗和静脉激素治疗。5例气管内着火病例的气道均无严重烧伤。

#### 2.1.3 随访情况

145例恶性肿瘤患者随访3个月-36个月，失访16例（11.0%），平均生存时间5个月。1个月内死亡率为13.1%（19/145），其中大出血死亡4例，其余均为呼吸衰竭等原因死亡。

### 2.2 良性狭窄的治疗效果

#### 2.2.1 首次治疗后气道阻塞程度和临床状况的变化

49例病例共进行硬质镜治疗76人次，平均每例（1.6±0.2）次。所有患者共进行支气管镜检查（包括硬质镜）669次，平均每例（12.2±2.1）次，硬质镜占11.4%。

病变部位：主气管51人次；右主支气管11人次，右中间段支气管8人次，右上叶支气管和右下叶支气管各1人次；左主支气管13人次，左下叶支气管2人次，左上叶支气管1人次。有4例患者同时有两个以上部位的病变。

对21例气道瘢痕狭窄病变首先采取APC，再结合冻取将管腔扩大，残留部位采用冻融（图 2）。其中4例患者放置被膜金属支架（2个直型，2个L型）。

**2:**
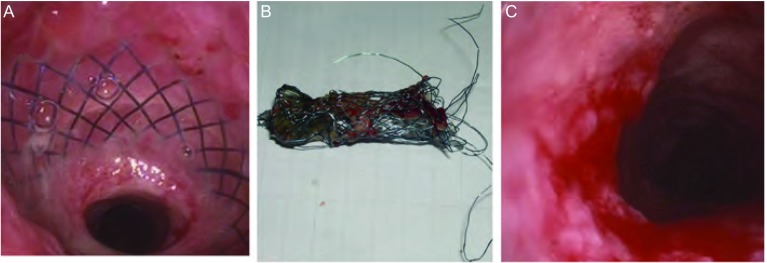
气管内支架置入后肉芽组织增生。A：气管上段可见网状裸支架，支架两端被肉芽组织包埋，下端管腔狭窄；B：取出的支架已破损、变形；C：取出支架后，管腔扩大，管壁上遗有肉芽组织。 Granulation tissue developed after airway stenting. A: Uncovered stent was discovered at the end of upper trachea accompanied with granulation and endoluminal strictor; B: Retrieved stent was destroyed and deformed; C: Endolumen was expanded and granulation was remained in tracheal wall after stent retrieval.

肉芽肿性病变中1例采用电圈套器将大的肉芽取出，残留部位采用APC联合冷冻的方法，其余8例均采取APC联合冻取，将肉芽组织清除。10例良性肿瘤均采用冻取联合APC的方法，将肿瘤取出。5例气道内异物中的2例在直视下将异物取出，3例先用APC将异物表面的肉芽清除，再用冻粘的方法将暴露的异物取出。3例气管软化患者均直接放置直筒型被膜金属支架。

19例因置入支架后肉芽组织增生引起的气道狭窄，均先用APC将支架表面的肉芽组织清除。18例将支架取出（图 2），其中取出裸支架11个，取出后均呈碎片状，2例患者气道内各放置2个支架均取出，9个被膜金属支架取出后均完整。1例裸支架因放置时间较长，家属不同意取出。

首次硬镜治疗后气道阻塞程度和气促指数均明显下降（表 3）。

**3:** 首次硬质镜治疗前后良性气道狭窄患者的病情变化 Changes of airway obstruction and clinical manifestations before and after first rigid bronchoscopy in patients with benign airway strictures

Observed index	*n*	Before	After	*t*	*P*
Airway obstruction (%)	81	67.8±2.3	19.2±1.8	16.6	< 0.001
Shortbreath score	77	2.4±0.1	1.2±0.1	8.5	< 0.01

#### 2.2.2 并发症及处理

76人次患者硬质镜治疗过程中56次（占73.7%）术中出现血氧饱和度低于90%，采取措施为暂停治疗、改接麻醉机快速直接充氧、手法控制呼吸直到血氧饱和度恢复至95%以上后继续治疗；治疗中有3例重症患者（其中2例术前一直使用呼吸机）需要持续应用麻醉机快速直接充氧手法控制呼吸，直至堵塞气道的狭窄清除后方改为高频喷射通气。2例气管内肉芽肿性病变术中发生大出血, 均经快速负压吸引后，通过APC和药物联合应用，将血止住，无1例术中死亡。

## 3 讨论

硬质气管镜（简称硬质镜）的应用已有100多年的历史，它能保持气道通畅，并且在操作端有侧孔与呼吸机相连，故硬质镜亦称“通气支气管镜”^[1]^。电视硬质镜的现代价值在于可作为介入通道允许软性支气管镜及其他器械进入气道内，大大拓宽了其应用范围，可在直视下进行支架释放、激光消融、氩等离子体凝固术、取异物和冷冻等操作^[2-4]^。

本文194例患者共接受了325次硬质镜检查，平均每例患者接受1.6次操作，均一次性插入成功。所有这些患者共进行了1, 525次气管镜检查，硬质镜的操作次数占21.3%。其中良性狭窄每例接受12.2次气管镜检查，恶性病变每例接受5.9次检查，良性病变接受气管镜的检查次数明显多于恶性者，但接受硬质镜的检查次数相当。可见这些病例经首次硬质镜治疗后，狭窄好转，后期操作单独应用电子支气管镜即可，大大减轻了患者的痛苦，降低了治疗风险。

对恶性气道狭窄的患者，一般难以平卧，在全麻下插入硬质镜，既可保证患者的通气，又可从容地进行各种操作。清除肿瘤的方法很多，如热消融（激光、微波、APC）、冻取、光学活检钳、电圈套器和硬质镜直接铲除等。具体采用哪些方法更合适，需考虑内镜技术的熟练程度、已有的设备条件等。如气管中上部位阻塞较重、基底较宽的肿瘤，适合硬质镜直接铲除（本文有5例采用此方法），然后快速用硬质活检钳将肿瘤取出，以免引起窒息；对有蒂或瘤体较长的肿瘤则适合用电圈套器或光学活检钳将肿瘤直接切除（本文有8例采用此方法）；对瘤体表面较脆、易出血的肿瘤则适宜先用APC封闭血管，再结合冷冻将肿瘤冻取（本文大部分采用此方法）；对瘤体较弥漫、不易出血的肿瘤，亦可直接用冻取的方法，必要时结合APC（本文有30例采用此方法）。通常是以硬质镜作为通道并保障通气，如果肿瘤位于主气管内，用各种硬质器械均可操作；如果肿瘤位于支气管内，最好结合电子支气管镜进行各种操作。

由于结合了电子支气管镜，硬质镜治疗无论对主气管还是支气管狭窄都是非常有效的，本文显示支气管阻塞的改善程度要优于主气管。本组病例约半数为复合气道病变（两个部位以上），其中主气管病变占2/3，这是进行硬质镜检查的主要适应症。经硬质镜首次治疗后气道阻塞程度和气促指数明显下降，而KPS评分明显提高，说明单次硬质镜操作即可明显改善患者的生存质量。

对阻塞性肺不张有明显疗效，特别是对全肺不张的治疗效果要明显优于肺段不张。本组20例全肺不张的患者，经硬质镜治疗后全肺复张55%，部分复张35%，只有10%未能复张。6例位于右中间段的肿瘤均全部清除，引起的中下叶肺不张均消失。而31例肺段不张的患者，经硬镜治疗后全部复张38.7%，部分复张22.6%，未能复张38.7%。本文术中发生1例致死性大出血，是因肿瘤侵蚀右中间段支气管，将肿瘤取出后血管破裂大出血。还有3例大出血发生于下叶背段开口活检时。还有2例大出血因肿瘤清理过度，将阻塞5级支气管内的肿瘤均清除后分别于术后1周、3周大出血死亡。另外，转移性肿瘤血管丰富，活检时也易引起出血。因此，对肺内的肿瘤勿求彻底清除，可结合药物注射、放/化疗粒子植入等方法，消灭残余肿瘤^[2]^。

本组病例除了在硬质镜下清除肿瘤，还取出气管支架5例，放置L型支架6例、植入放/化疗粒子6例、光动力治疗（photodynamic therapy, PDT）3例。对某些危重患者，全麻下取出或放置支架更为安全^[5, 6]^。硬质镜下PDT主要用于3例咽喉部肿瘤的治疗，由于肿块严重堵塞声门部，局麻下难以进行治疗，则首先在全麻下将硬质镜前段骑跨在声门上，一边通气，一边治疗，很快将肿瘤清除。传统认为硬质镜适应于声门下2 cm的肿瘤，实际上声门下气管高位肿瘤同样可应用，且比较安全、有效。特别是在PDT过程中，硬质镜前段骑跨声门上，同时可有效保护对侧声门，避免PDT引起的声门水肿。

对良性气道狭窄，硬质镜同样安全、有效。首次硬质镜治疗后，气道阻塞程度和气促指数均明显降低。患者经过初次硬质镜治疗，气道梗阻症状大大缓解后即可在局麻下进行后期治疗。气管插管或气管切开后的病变主要在气管上段，而结核病变大多位于左主支气管。单纯球囊扩张易复发，本文先采用APC热消融的方法，消除狭窄的病变，再结合冻融的方法，防止狭窄复发。对放置支架后形成的肉芽肿，应将支架及时取出，再采取冻融等方法，切不可在狭窄的部位再摞置支架。对气管软化或复发性多发性软骨炎的患者，放置被膜金属支架是最佳选择。本组3例气管软化的患者放置支架1个月-2个月后，2例将支架取出，再结合冻融的方法，半年后均治愈，1例气管切开、气管软化的患者放置支架1个月后死于大出血；1例复发性多发性软骨炎的患者已放置Y型被膜金属支架5年仍存活，其中有两次曾因支架两端有肉芽形成而行气管镜下治疗，支架一直保留在气道内。

气道内大的良性肿瘤或息肉可采取气道内恶性肿瘤类似的治疗手段，将病变组织清除。

作者还采用先清肉芽、后碎支架，然后将支架单丝抽出的方法，将长期滞留在气管内的11个金属裸支架取出（包括1例放置7年的支架），后再联合APC及冷冻的方法最终治愈。9个可回收型被膜金属支架的7例则被完整取出，1例回收线断裂，用光学活检钳取出，还有1例破碎的被膜支架也用光学活检钳逐一取出。1例因裸支架放置时间较长，家属拒绝取出。本文还有7例患者在硬质镜下成功放置被膜金属支架（5个直筒型，2个L型），其中2例术前一直使用呼吸机，放置支架后均停用呼吸机。

硬质镜操作过程中比较安全。持续APC烧灼易引起低氧血症^[7]^。本组约75%发生术中缺氧，但停止操作并给氧后，血氧饱和度会很快上升，恢复至正常后可继续操作。对引起气道严重堵塞的肿块应尽快清除，以防窒息。气道内发生大出血时，应持续负压吸引，勿使血凝块堵塞气道，必要时换用双腔气囊导管或肺动脉栓塞止血。本文尚未采用这些方法。无1例严重缺氧死亡。本组虽有5例发生烧毁内镜的事件，但如处理及时对患者并无大的风险。APC烧灼过程中切勿长时间持续操作，可避免此类事件发生。

当然，硬质镜操作需熟练的麻醉科医师密切配合^[8]^。辅助性机械通气要求保留患者的部分自主呼吸，特别是气道堵塞严重、呼吸困难的患者，更应严格掌握剂量，以免引起心脏功能抑制和血压下降。控制性机械通气是将患者自主呼吸完全控制，一般辅以肌松剂，适用于身体状况较好、气道反射性很强的患者。操作一段时间后，可能会引起CO_2_潴留，应将硬质镜后孔封闭，启用手动式球囊按压，促进排气。自主呼吸一般是在手术快要结束时，停止输注静脉麻醉药，待患者自主呼吸完全恢复、血氧饱和度维持在95%以上时可将硬质镜拔出。但全身静脉麻醉需在手术室进行，费用较高，术后还需恢复过程，这是硬质镜的主要缺点。
